# Identification of deep intronic variants of *PAH* in phenylketonuria using full-length gene sequencing

**DOI:** 10.1186/s13023-023-02742-1

**Published:** 2023-05-26

**Authors:** Chuan Zhang, Yousheng Yan, Bingbo Zhou, Yupei Wang, Xinyuan Tian, Shengju Hao, Panpan Ma, Lei Zheng, Qinghua Zhang, Ling Hui, Yan Wang, Zongfu Cao, Xu Ma

**Affiliations:** 1 Gansu Province Medical Genetics Center, Gansu Provincial Maternity and Child-Care Hospital, Lanzhou, China; 2 National Research Institute for Health and Family Planning, National Human Genetic Resources Center, Beijing, China; 3 Graduate School of Peking, Union Medical College, Beijing, China; 4 Prenatal Diagnostic Center, Beijing Obstetrics and Gynecology Hospital, Capital Medical University; Beijing Maternal and Child Health Care Hospital, Beijing, China

**Keywords:** PKU, *PAH*, Deep intronic variant, RNA splicing, Minigene

## Abstract

**Background:**

Phenylketonuria (PKU) is an autosomal recessive congenital metabolic disorder caused by *PAH* variants. Previously, approximately 5% of PKU patients remained undiagnosed after Sanger sequencing and multiplex ligation-dependent probe amplification. To date, increasing numbers of pathogenic deep intronic variants have been reported in more than 100 disease-associated genes.

**Methods:**

In this study, we performed full-length sequencing of *PAH* to investigate the deep intronic variants in *PAH* of PKU patients without definite genetic diagnosis.

**Results:**

We identified five deep intronic variants (c.1199+502A>T, c.1065+241C>A, c.706+368T>C, c.706+531>C, and c.706+608A>C). Of these, the c.1199+502A>T variant was found at high frequency and may be a hotspot *PAH* variant in Chinese PKU. c.706+531T>C and c.706+608A>C are two novel variants that extend the deep intronic variant spectrum of *PAH*.

**Conclusion:**

Deep intronic variant pathogenicity analysis can further improve the genetic diagnosis of PKU patients. In silico prediction and minigene analysis are powerful approaches for studying the functions and effects of deep intronic variants. Targeted sequencing after full-length gene amplification is an economical and effective tool for the detection of deep intron variation in genes with small fragments.

## Introduction

Hyperphenylalaninemia (HPA) is the most common hereditary disorder of amino acid metabolism worldwide. The main type of HPA is phenylketonuria (PKU), an autosomal recessive disease caused by variants of *PAH*, the gene encoding phenylalanine hydroxylase [24]. As of November 2, 2022, the PAHvdb database (http://www.biopku.org/home/pah.asp) has collected 1583 types of *PAH* variants, including missense, frameshift, synonymous, and splicing variants, UTR variation, and large-scale deletion. These variations are mainly concentrated in exons and exon–intron boundaries. Existing molecular detection technology can accurately detect the above *PAH* variants; however, in different regions of the world, regardless of the detection technology applied, the genetic diagnosis rate of PKU by researchers is 70.6–96%, which fails to reach 100% [3, 4, 8, 14–16, 20, 22, 27, 28, 30]. Therefore, sequence information limited to exons and exon–intron boundaries cannot identify the entire genetic information of PKU.

Human protein-coding genes consist, on average, of short coding fragments containing 8–10 exons that are interrupted by noncoding sequences or introns that are approximately 20 times longer [27]. Introns have been critical for the evolution of eukaryotes. The intron–exon structure of eukaryotic genes plays an important role in the generation of new genes through exon shuffling [5, 19], and the ability to alternately select different exon combinations is crucial to the gene expression diversity of complex organisms [13]. With the clinical application of Whole Genome Sequencing (WGS) technology, more and more pathogenic deep intron variants (more than 100 bp from the exon–intron boundary) have been discovered. Variants in deep intronic regions exist in a variety of diseases, and hundreds of studies on the pathogenesis of deep introns have been published. Deep intron mutations are located at least 100 bp away from the nearest typical splice site [27]). Therefore, the pathogenicity analysis of deep intron variants of *PAH* may be a new strategy to improve the diagnostic rate of PKU when only one pathogenic variant is found in exons and flanking sequences of *PAH*.

We previously identified three *PAH* deep intron variants (c.706+368T>C, c.1065+241C>A, and c.1199+502A>T) in ten patients with PKU in northwest China using WGS [12]. Later, Gao et al. [10] also found deep intronic *PAH* variants in patients with PKU without definite genetic diagnosis, which indicated that the study of deep intronic variation of *PAH* can improve the PKU genetic diagnosis rate. Therefore, in this study, we performed full-length sequencing of *PAH* for PKU patients with unknown genotypes to investigate whether they were carrying pathogenic deep intronic *PAH* variants, and to expand the pathogenic variation spectrum of deep intronic variation of *PAH*.

## Methods

### DNA samples

**Fig. 1:**
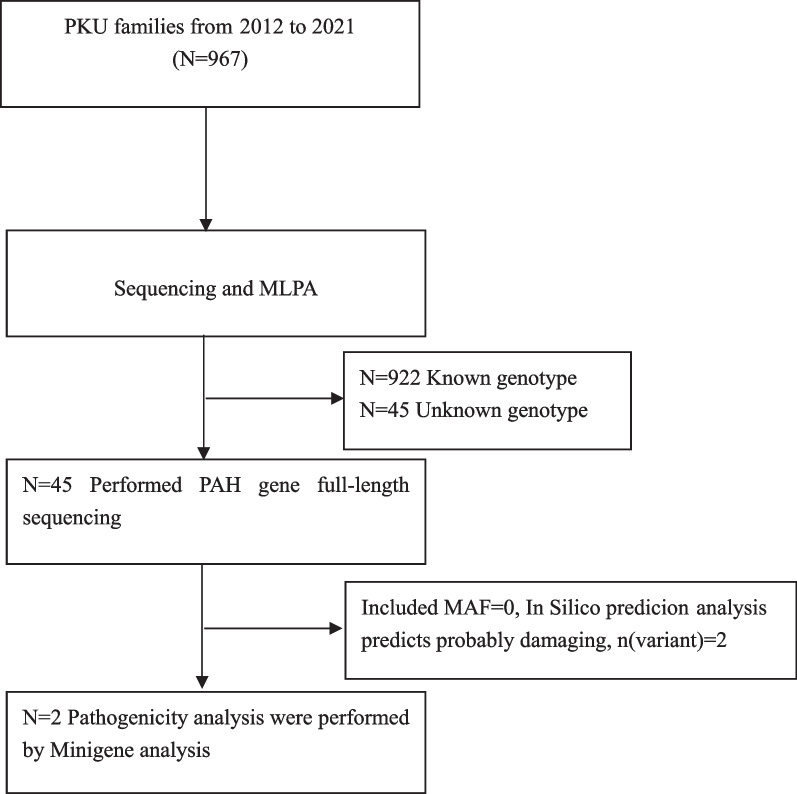
Flow diagram of the recruitment and exclusion process for the PKU patients

Based on clinical features and newborn screening, 967 cases of PKU were diagnosed in the Medical Genetics Center of Gansu Provincial Maternity and Child-Care Hospital between January 2012 and December 2021. From these patients, we selected 45 patients with only one or no variants detected in *PAH*, and screened for *PAH* variants by Sanger sequencing, whole exon sequencing, and MLPA on diagnosis (Fig. 1). Of these 45 patients, according to the standard classification criteria [29], 11 were classic PKU (cPKU, ≥ 1200 µmol/L), 17 were mild PKU (mPKU, 360–1200 µmol/L), and 17 were mild hyperphenylalaninemia (MHP, 120–360 µmol/L). This study was undertaken according to the tenets of the Declaration of Helsinki 1975 and its later amendments. The study protocol was approved by the Ethics Committee of the Gansu Provincial Maternity and Child-Care Hospital (2021GSFY[65]). Written informed consent was obtained from all study participants or their legal guardians.

### Genomic DNA preparation

Genomic DNA was extracted from peripheral blood samples (2–3 mL) of the probands and their parents using the Tiangen DNA extraction kit (Tiangen Biotech, China). DNA quality was quantified with a NanoDrop 2000 (Thermo, USA).

### Full-length sequencing of PAH

We designed 13 pairs of primers to cover the whole *PAH* gene using Primer3 online software (v.0.4.0, https://bioinfo.ut.ee/primer3-0.4.0/), the primers and fragment length of Full-length amplification of *PAH* were showed in Table 1. The PCR-amplified fragments were tested using 1% agarose gel electrophoresis. After successful amplification, the products were mixed and sent to Beijing Nuo Zhiyuan Technology Co., Ltd for targeted sequencing. The library for genome sequencing was generated from *PAH* full-length amplified products using the Illumina TruSeq DNA PCR-Free Library Prep Kit (Illumina). Sequencing was performed on the Illumina HiSeq 2500 System, the sequencing range covered the entire *PAH* gene, and the sequencing depth was 10,000X. Data analysis and variant curation were performed using Pgenomics software (https://www.pgenomics.cn/). Single-nucleotide variants and small insertions and deletions were identified using MedGAP v.2.0, a pipeline based on GATK best practices for data preprocessing and variant discovery with the GATK Haplotype Caller (https://gatk.broadinstitute.org/hc/en-us, v.3.1.1) [12].

**Table 1:** The primers and fragment length of Full-length amplification of *PAH*

Fragment	Primers	Primer sequence 5′–3′	Genomic locus	Fragment length
1	PAH-L1F	ACTCTCTTCTCCTCCCTAGTGCG	chr12:103311000	11,916 bp
	PAH-L1R	CCAAATAGCTCCCTGATTCACCC	chr12:103299084	
2	PAH-L2F	AAGGTAGACAAGGTGGTAGGACT	chr12:103299364	3975 bp
	PAH-L2R	GAGAGCACACTTCAAAAAGAAGG	chr12:103295389	
3	PAH-L3F	TGCTTTTCCCACTGTTACACTCC	chr12:103295660	4029 bp
	PAH-L3R	GGCTGGATGACTCAAGAGTTTTA	chr12:103291631	
4	PAH-L4F	ACGAATGTGGGAGTGGGATGCTT	chr12:103291906	3144 bp
	PAH-L4R	ACCAGAACAGGAAAACCTAACGC	chr12:103288762	
5	PAH-L5F	GTCTGACCCCCTATTCAAGCAGA	chr12:103289098	11,693 bp
	PAH-L5R	ACCTCTGAGCTCTGCACCTTGTC	chr12:103277405	
6	PAH-L6F	CCCATCAACCCTCTGAAGGACGT	chr12:103277697	10,531 bp
	PAH-L6R	TGAAGGAGGGTACAGCCATTGGT	chr12:103267166	
7	PAH-L7F	TCGTGAGTGGTAGTTTTCCATGG	chr12:103267491	5388 bp
	PAH-L7R	AAGGAAGGGAGGGAAGATAGGAG	chr12:103262103	
8	PAH-L8F	TAGAAACGAGGCACAACAGTAGT	chr12:103262383	2337 bp
	PAH-L8R	AGGCTGTTTTATTCAGGACCGAG	chr12:103,260,046	
9	PAH-L9F	CCTGTGTACCGTGCAAGACGGAA	chr12:103260407	8101 bp
	PAH-L9R	TCTCACCACATTGCACTCATTCC	chr12:103,252,306	
10	PAH-L10F	CCTCTACACACTGCCTTAAATGT	chr12:103252590	4048 bp
	PAH-L10R	ACACAAACACACACTCCTAACTC	chr12:103248542	
11	PAH-L11F	GGAAGACAAGTATGTGGAGGCAA	chr12:103,248,822	4049 bp
	PAH-L11R	GATTGTTTGAGCCCAGAAGTCTG	chr12:103244773	
12	PAH-L12F	CCTTGTCAGGCAGTTATTTGTGT	chr12:103245076	3913 bp
	PAH-L12R	ATAGCTGGAGAGTCTAACACATC	chr12:103,241,163	
13	PAH-L13F	CCCTGGAGCTCTTAGTCCCTCTTGTTT	chr12:103241472	10,642 bp
	PAH-L13R	AGCAAGATCATCTGTCAGTAAAGACTG	chr12:103230830	

Variants were described according to the nomenclature recommended by the Human Genome Variation Society (www.hgvs.org/). Variant frequencies were searched in the GnomAD (http://gnomad.broadinstitute.org/), Exome Sequencing Project (ESP, http://evs.gs.washington.edu), and dbSNP (http://www.ncbi.nlm.nih.gov/projects/snp) databases. Suspicious variants were verified by Sanger sequencing, and the PCR products were bidirectionally sequenced using the BigDye Terminator v3.1 Cycle Sequencing Kit (Applied Biosystems, USA) on an ABI 3500DX Genetic Analyzer (Applied Biosystems) after purification on 2% agarose gels.

### In silico* prediction of deep intronic variants*

Alamut Visual v.2.11 (Interactive Biosoftware) software was used to predict the influence of variation sites on splicing site selection. The process included a splicing module that integrates a number of prediction algorithms and splicing prediction data using Splice Site Finder-like (SSFL), MaxEntScan (MES), NNSplice, and GeneSplicer to analyze splicing signals. Exonic splicing enhancer (ESE) finder 3.0 and RESCUE-ESE were used for ESE binding site prediction and for high confidence branch point prediction [12]. Individual tools were deemed to predict altered splicing where the change in the splice site score was ≥ 10% (based on MES and GeneSplicer) or ≥ 5% (based on NNSplice and SSFL). SVM-BPfinder (http://regulatorygenomics.upf.edu/Software/SVM_BP/) and RNABP: Branch Point Selection in RNA Splicing Using Deep Learning (http://nsclbio.jbnu.ac.kr/tools/RNABP/) were used to predict the RNA splicing branch points.

### Minigene analysis

To evaluate the in vitro splicing of two deep intronic variants (c.706+531T>C and c.706+608A>C) in *PAH*, we constructed minigenes using the pMini-CopGFP vector (Invitrogen). In the minigene pMini-CopGFP (+) vector, _In6 for c.706+531T>C and c.706+608A>C (a fragment of the human *PAH* gene including full-length exon 6, intron 6, and exon 7) were amplified from the patient’s genomic DNA using primers located in introns 5 and 7. The gene fragments and their flanking regions were cloned into pMini-CopGFP (+) using BamHI/XhoI restriction endonucleases. The mutant and wildtype minigene constructs were thus prepared. The positive cloning screening/identification primers for the two sites were the same: β-globin intron-F, 5′-GATATACACTGTTTGAGATGAGGA-3′; PAH-E6-E7-R, 5′-TAGATATGCTACTAATCCCC-3′.

For the minigene assays[12], 293T cells were seeded in 35 cm^2^ wells (density, 2–3 × 10^5^) in 2 mL of 10% minimum essential medium (MEM) and then grown overnight. Cells were transfected with DNA (4 µg per well) using Lipofectamine 3000 Transfection Reagent (Thermo Fisher). Cells were harvested by trypsinization after 48 h. Total RNA was isolated using TRIzol Reagent (Thermo Fisher) and phenol–chloroform extraction. Complementary DNA (cDNA) synthesis was performed using the HiScript II 1st Strand cDNA Synthesis Kit (+ gDNA wiper) (Vazyme). Splicing analysis of the two intronic variants was performed by PCR amplification with FastStart Taq Polymerase (Vazyme) using the specific primers F: 5′-GGCTAACTAGAGAACCCACTGCTTA-3ʹ, and R: 5ʹ-GGTTCGGGGGTATACATGGGCTT -3ʹ for the pMini-CopGFP (+) minigene. PCR products were confirmed by Sanger sequencing after purification on 2% agarose gels.

## Results

### Screening for deep intronic variants

Of the 967 PKU patients, only 45 patients showed a heterozygous variant in the *PAH* gene after exon or exon–intron junction detection of the *PAH* gene. All the 45 patients got successfully full-length sequencing, sequencing quality shows an average sequencing depth of 9019× (ranged 3731× to 15,097×). After full-length sequencing of *PAH*, 24 of 45 PKU patients showed pathogenic and suspected pathogenic intronic variants, including 11 cPKU, 11 mPKU, and two MHP (Table 2). No suspected disease-causing variants had been identified for six mPKU and 15 MHP patients (Table 2). Among the 24 PKU patients with definite genotyping, 18 patients had the c.1199+502A>T variant, three patients had c.1065+241C>A, one patient had c.706+368T>C, one patient carried c.706+531T>C, and one carried c.706+608A>C. The variants c.1199+502A>T, c.1065+241C>A, and c.706+368T>C were classified as likely pathogenic or pathogenic in our previous study [16], and c.706+531T>C and c.706+608A>C were two novel variants that had not previously been reported.

**Table 2:** Genotypes of 45 undiagnosis PKU families

ID	Classifcation	Paternal variant	Maternal variant
1	cPKU	c.728G>A	c.1199+502A>T
2	cPKU	c.1197A>T	c.706+368T>C
3	cPKU	c.1199+502A>T	c.728G>A
4	cPKU	c.1199+502A>T	c.728G>A
5	cPKU	c.1301C>A	c.1065+241C>A
6	cPKU	c.1199+502A>T	c.1238G>C
7	cPKU	c.1238G>C	c.1199+502A>T
8	cPKU	c.1199G>A	c.1199+502A>T
9	cPKU	c.1199+502A>T	c.728G>A
10	cPKU	c.1199+502A>T	c.782G>A
11	cPKU	c.526C>T	c.1199+502A>T
12	mPKU	c.1199+502A>T	c.1199G>A
13	mPKU	c.208_210delTCT	c.1065+241C>A
14	mPKU	c.1199+502A>T	c.694C>T
15	mPKU	c.842+2T>A	c.1199+502A>T
16	mPKU	c.1068C>A	c.1199+502A>T
17	mPKU	c.1199+502A>T	c.194T>C
18	mPKU	c.1199+502A>T	c.331C>T
19	mPKU	c.87C>A	c.1199+502A>T
20	mPKU	c.1065+241C>A	c.728G>A
21	mPKU	EX1-2del	c.706+531T>C
22	mPKU	c.706+608A>C	c.694C>T
23	mPKU	?	c.782G>A
24	mPKU	c.1301C>A	?
25	mPKU	c.782G>A	?
26	mPKU	c.1289T>C	?
27	mPKU	?	c.473G>A
28	mPKU	c.1301C>A	?
29	MHP	c.1199+502A>T	c.158G>A
30	MHP	c.532G>A	c.1199+502A>T
31	MHP	Exon3del	?
32	MHP	EX1-Updel	?
33	MHP	c.1256A>G	?
34	MHP	?	c.770G>T
35	MHP	c.1114A>T	?
36	MHP	c.611A>G	?
37	MHP	c.728G>A	?
38	MHP	c.194T>C	?
39	MHP	?	c.1200-1G>C
40	MHP	?	c.1068C>A
41	MHP	?	c.1068C>A
42	MHP	c.1315+6T>A	?
43	MHP	c.688G>A	?
44	MHP	?	c.782G>A
45	MHP	c.208_210delTCT	?

### In silico* prediction analysis*

The novel deep intronic variant c.706+531T>C was identified in a patient with cPKU, where this variant formed a compound heterozygous mutation with EX1-2del in the proband. The novel deep intronic variant c.706+608A>C was also identified in a patient with cPKU, this variant formed a compound heterozygous mutation with c.694C>T in the proband. Both variants were absent in the ClinVar (www.ncbi.nlm.nih.gov/clinvar/), HGMD (www.hgmd.cf.ac.uk), and gnomAD (http://gnomad.broadinstitute.org/) databases.

**Fig. 2:**
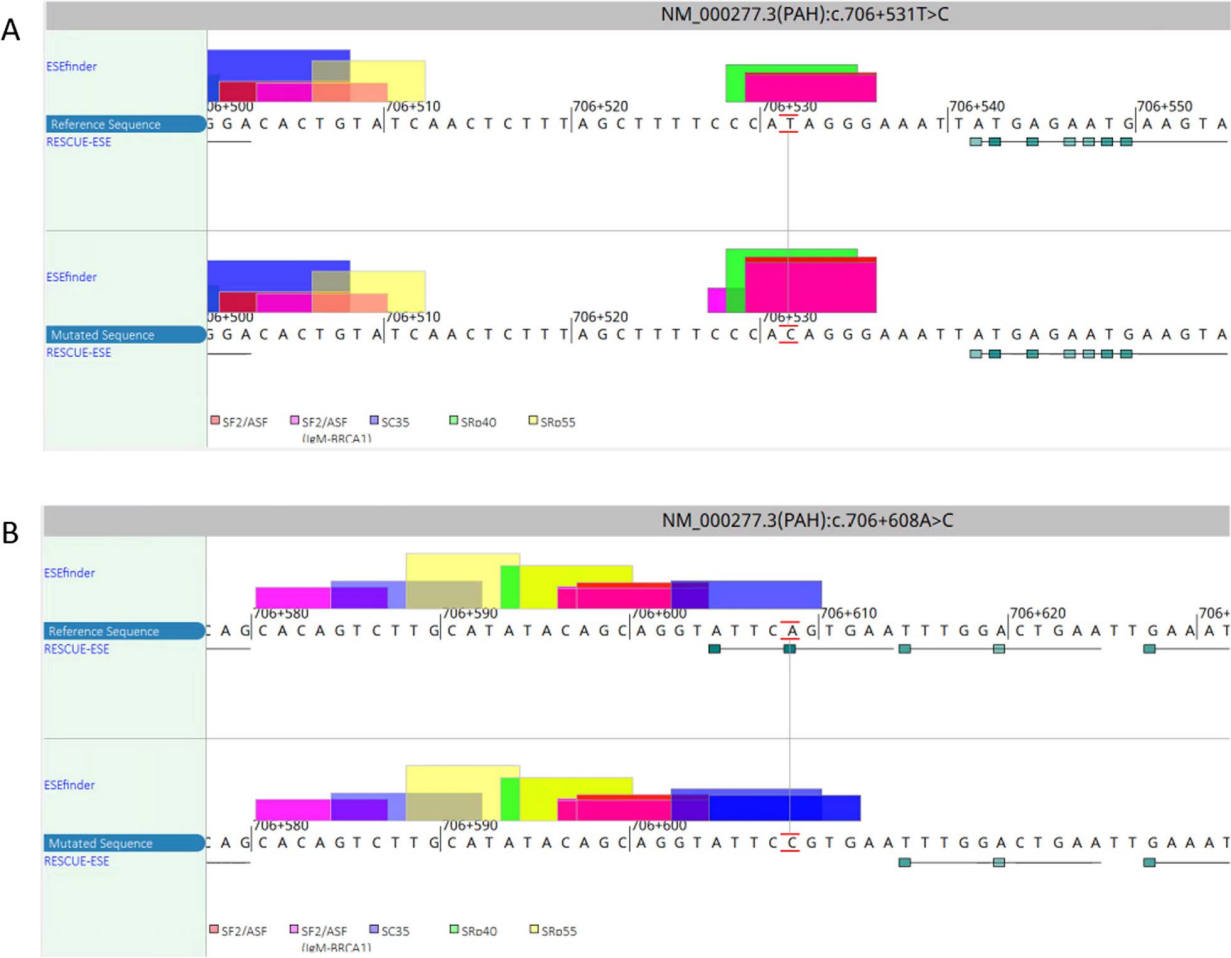
In Silico prediction of deep intronic variants. **A** ESEfinder predicted that the c.706+531T>C might create an SRSF1 (SF2/ASF(IgM-BRCA1)) binding sites; **B** ESEfinder predicted that the c.706+608A>C variant may strengthen the SRSF2 (SC35) binding sites

In silico analysis by Alamut Visual predicted that the two variants probably impact splice site selection (Table 3 and Fig. 2). Using ESEfinder, c.706+531T>C was predicted to create an SRSF1 (SF2/ASF(IgM- BRCA1)) binding site and c.706+531T>C was predicted to strengthen the SRSF2 (SC35) binding site.

**Table 3:** In silico prediction scores for the splice sites of two deep intronic variants assessed in this study by Alamut^®^ Visual

Variant	Position	3′/5′	SSFL (0–100)		Max EntScan (0–12)		NNSPLICE (0–1)		GeneSplicer (0–21)		Branch points (0–100)	
			WT	Mut	WT	Mut	WT	Mut	WT	Mut	WT	Mut
c.706+531T>C	c.706+534	3′	–	-	7.6	8.1	1.0	1.0	–	–	–	–
	c.706+530	3′	–	–	–	–	–	–	–	–	67.7	70.9
	c.706+532	3′	–	–	–	–	–	–	–	–	52.7	59.3
c.706+608A>C	c.706+601	5′	–	–	–	–	0.7	0.8	–	–	–	–
	c.706+613	3′	–	–	–	–	–	–	–	–	66.5	69.7
	c.706+614	3′	–	–	–	–	–	–	–	–	46.3	48.8

The higher the score, the higher the credibility, “–” no predictive splicing sites

### Minigene analysis

**Fig. 3:**
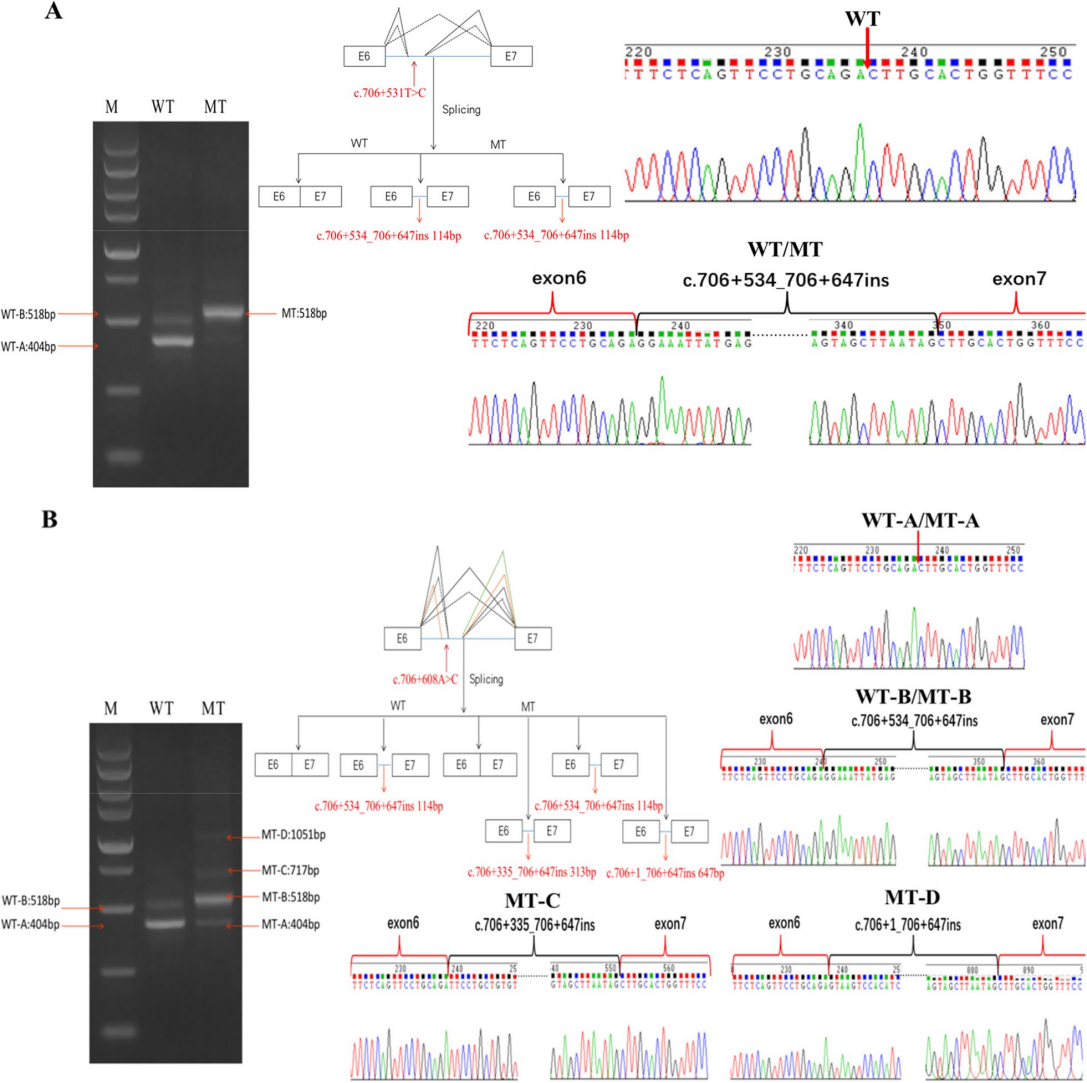
Variant splice effects seen by minigene RT-PCR analysis. **A** The c.706+531T>C whild type minigene produced a major fragment (WT-A) and one low abundance fragments (WT-B), mutant minigene only produced a fragment (MT). Sanger sequencing showed that WT-B and MT include a 114-nt pseudo-exon upstream of exon 7. **B** The c.706+608A>C whild type minigene produced a major fragment (WT-A) and one low abundance fragments (WT-B), mutant minigene produced a major fragment (MT-B) and three low abundance fragments (MT-A, MT-C, MT-D). Sanger sequencing showed that WT-B and MT-B include a 114-nt pseudo-exon, MT-C includes a 313-nt pseudo-exon and MT-D includes a 647-nt pseudo-exon upstream of exon 7. RT-PCR, reverse transcription polymerase chain reaction

The RT-PCR results of the c.706+531T>C variant showed that there were two amplified bands in the WT(whild type) group and only one in MT(mutant type), among which only 495 bp of the expected sequence was amplified in the WT group. In addition, both groups had 518 bp bands, but the WT group had relatively weak bands (Fig. 3). Sanger sequencing showed that the WT-A band was consistent with the expected transcript sequence and the normal splicing mode, and was the main product of the WT group (Fig. 3A). The WT-B and MT bands resulted from the inclusion of a 114 bp intronic sequence (pseudo-exon) in intron 6. The splicing mode of the mRNA was NM_000277.3: c.706+534_706+647ins 114 bp (Fig. 3A). Therefore, it was classified as a likely pathogenic (PS1, PM3, PP3, and PP4) variant.

The RT-PCR results of the c.706+608A>C variant showed that there were two amplified bands in the WT group and four amplified bands in the MT group, where both 404 bp and 518 bp fragments were amplified in both the WT and MT groups. In addition, the WT group had a relatively weak 518 bp band while the MT group had a relatively weak 404 band (Fig. 3B). The MT group also had two relatively weak bands (MT-C and MT-D, Fig. 3B). Sanger sequencing showed that the WT-A band was consistent with the expected transcript sequence and the normal splice mode, and was the main product of the WT group (Fig. 3B). The WT-B and MT-B bands resulted from the inclusion of a 114 bp intronic sequence (pseudo-exon) in intron 6. The mRNA splicing mode was NM_000277.3: c.706+534_706+647ins 114 bp (Fig. 3B). The MT-C band resulted from the inclusion of a 313 bp intronic sequence (pseudo-exon) in intron 6, and the splicing mode of the mRNA was NM_000277.3: c.706+335_706+647ins 313 bp (Fig. 3B). The MT-D band resulted from the inclusion of a 647 bp intronic sequence (pseudo-exon) in intron 6, and the mRNA splicing mode was NM_000277.3: c.706+1_706+647ins 647 bp (Fig. 3B). Therefore, the pathogenicity of c.706+608A>C was classified as likely pathogenic (PS1, PM3, PP3, and PP4).

## Discussion

PKU is an autosomal recessive genetic disease, which is mainly caused by variation of the *PAH* gene, encoding phenylalanine hydroxylase. Early, rapid, and accurate genetic etiological analysis is very important for subsequent patient treatment, genetic counseling, and fertility guidance [10]. Previous studies on *PAH* gene variation have mainly focused on exon and flanking intron sequences using Sanger sequencing and multiplex ligation-dependent probe amplification (MLPA), resulting in an unsatisfactory genetic diagnosis rate for PKU [3, 4, 8, 14–16, 20, 22, 27, 28, 30]. Approximately 5% of PKU patients with typical clinical symptoms do not receive a definitive genetic diagnosis [18].

Introns are very important for eukaryotic evolution. The intron–exon structure of eukaryotic genes plays an important role in the generation of new genes through exon changes [5, 19], and the ability to alternately select different exon combinations is crucial for the gene expression diversity of complex organisms [13]. Nowadays, NGS has greatly improved the diagnosis of genetic diseases, and increasing numbers of pathogenic deep intronic variants have been discovered. Pathogenic deep intronic variants have been reported in more than 100 disease-associated genes. These pathogenic deep intronic variants commonly result in pseudo-exon inclusion via activation of atypical splice sites or changes in splicing regulatory elements [12, 27].

In this study, we performed pathogenicity analysis of deep intronic variants of the *PAH* gene in 45 patients with PKU, and we identified five different deep intron variants, c.1199+502A>T, c.1065+241C>A, c.706+368T>C, c.706+531T>C, and c.706+608A>C. Of these, c.1199+502A>T was detected in 18 patients, c.1065+241C>A was detected in three patients, and c.706+368T>C, c.706+531T>C, and c.706+608A>C were detected in one patient each. The c.1199+502A>T variant might be a hotspot of deep intronic variants for PKU patients in China. These results suggest that deep intronic variants in *PAH* can result in PKU, and *PAH* full-length sequencing can be used to identify deep intronic variants as well as WGS, with a lower detection cost.c.706+531T>C and c.706+608A>C in *PAH* are two novel variants that have not been reported previously. In silico prediction analyses showed that the two deep intronic variants may impact splice site selection, resulting in pseudo-exon inclusion in PAH. It has been reported that the deep intron variants can cause pseudo-exon inclusion [12]. The mechanism may involve the intronic variant creating a new donor splice site and activating a pre-existing atypical acceptor splice site [7, 21, 26]. Some studies have also reported that deep intronic variants can create a new acceptor splice site or interfere with the splicing of regulatory elements, which results in pseudo-exon inclusion [1, 9, 25].

The Alamut Visual software indicated that c.706+531T>C might impact splice site selection (Table 3) and affect the branch point (Table 4). This variant was predicted by ESEfinder to create an SRSF1 (SF2/ASF(IgM-BRCA1))-binding ESE in the variant sequence, leading to activation, probably by assisting with recognition of the weak splice donor site (SDS) in a similar way as previously reported [11, 23]. We identified a 114-nt pseudo-exon in the mutant type by RT-PCR analysis of the minigene. A deep intronic variant has previously been shown to create an SRSF1-binding ESE, leading to pseudo-exon activation [11, 23]. The wildtype intron 6 also produced 114-nt pseudo-exons, however, the bands were weak. We speculated that c.706+531T>C led to a stronger SRSF1 binding site (score: 4.76, Table 4), thus activating ESE. Another reason could be that c.706+531T>C affects the splice branching point sequence, but further experimental evidence is needed to verify this.

**Table 4:** In silico prediction scores for the c.706+531T>C and c.706+608A>C variants, as assessed by ESEfinder3.0

Variant	Position	SF2/ASF		SF2/ASF (IgM-BRCA1)		SC35		SRp40		SRp55	
		WT	mut	WT	mut	WT	mut	WT	mut	WT	mut
c.706+531T>C	CA***T***AGGG	2.68	5.29	2.69	4.76	–	–	–	–	–	–
	CCA***T***AGG	–	–	–	–	–	–	3.58	5.97	–	–
c.706+608A>C	–	–	–	–	–	–	–	–	–	–	–

WT: whild type; mut: mutant type; The threshold: SF2/ASF,1.956; SF2/ASF (IgM-BRCA1), 1.867; SC35, 2.383; SRp40, 2.67; SRp55, 2.676。 “–” indicates an unrecognized ESE sites

Alamut Visual software indicated that c.706+608A>C might impact splice site selection (Table 3) and affect the branch point (Table 4). Although ESEfinder predicted that c.706+608A>C would not change the ESE (Table 4), it might strengthen the SRSF2 (SC35) binding site. We identified 114 nt, 313 nt, and 647 nt pseudo-exons in the mutant type by RT-PCR analysis of the minigene. Although the wildtype intron 6 also produced 114 nt pseudo-exons, the bands were also weak. Similar to c.706+368T>C, we hypothesized that although the c.706+608A>C variant creates a stronger SDS that is inhibited by TDP-43 binding, the c.706+608A>C variant might also activate a cryptic SDS and splice acceptor site (SAS) by strengthening an SRSF2 (SC35) binding site, resulting in pseudo-exon generation [12]. However, as before, whether this is because it affects the splicing branch point sequence still requires further experimental evidence for verification.

Jin et al. [12] performed WGS in 10 undiagnosed PKU patients and identified three pathogenic deep intronic variants. In this study, we, In this study, we used 13 pairs of primers to amplify the whole *PAH* gene, and we found two pathogenic deep intronic variants through targeted full-length sequencing of *PAH*. WGS can effectively detect deep intronic variant, and has been widely used in the diagnosis of genetic diseases. However, WGS also has certain defects, such as low sequencing depth and high price. The full-length sequencing of *PAH* used in this study to detect deep intronic variant achieves the same effect as WGS, with an average sequencing depth of 9019×, and the detection cost is only 20% of WGS. Therefore, targeted sequencing after amplification of the entire gene can be an economical and effective strategy to study the deep intronic variation of the target gene.

Pathogenicity analysis of deep intronic variant in *PAH* can improve the genetic diagnosis rate of PKU. However, three mPKU and 15 MHP cases in our study did not have a clear genetic diagnosis. Gao et al. also found three mPKU cases and one cPKU case without a definitive genetic diagnosis after detecting deep intron variants in the *PAH* gene [10]. This could be because some non-*PAH* genes may affect *PAH* function and subsequently increase blood phenylalanine concentrations [2], and may also be related to some epigenetic factors [6]. The lncRNA *Pair* and the human *HULC* gene are associated with *PAH* and modulate enzymatic activities by facilitating *PAH* substrate and *PAH*–cofactor interactions [10, 17]. In addition, existing studies on deep intronic variants mainly analyzed that missense variant, whether indel variant or rearrangement of deep intron region also affect the function of *PAH* needs further investigation.

## Conclusions

In summary, we analyzed the pathogenicity of deep intron variants in the *PAH* gene, and pathogenic deep intronic variants were identified in 24 of 45 PKU patients with unknown genotypes. We identified two novel pathogenic deep intronic variants in *PAH*, extending the deep intronic variant spectrum of *PAH*. This study has shown that deep intronic variant pathogenicity analysis can further improve the genetic diagnosis of PKU patients, and suggests that pathogenic deep intron variation may not be uncommon in *PAH*. In silico prediction and minigene analysis can be powerful approaches for studying the functions and effects of deep intronic variants. Analysis of deep intron variation of disease-specific genes can be conducted by targeted sequencing after full-length gene amplification, and so this study provides a new avenue for the detection of deep intron variation in genes with small fragments.

## Data Availability

The data that support the findings of this study is available upon reasonable request from corresponding authors. The two novel variants have been submitted to the Clinvar (https://www.ncbi.nlm.nih.gov/ clinvar/). The accession numbers of c.706+531T>C was SCV002599130 and c.706+608A>C was SCV002599425.

## Abbreviations

cPKU: Classic PKU
ESE: Exonic splicing enhancer
HPA: Hyperphenylalaninemia
MHP: Mild hyperphenylalaninemia
MLPA: Multiplex ligation-dependent probe amplification
mPKU: Mild PKU
MT: Mutant type
PKU: Phenylketonuria
SAS: Splice acceptor site
SDS: Splice donor site
WGS: Whole genome sequencing
WT: Whild type
